# The impact of a personality trait rooted in Chinese confucian culture—Junzi personality—on loneliness and subjective well-being among chinese individuals: the mediating role of self-reflection and self-control

**DOI:** 10.1186/s40359-025-02488-4

**Published:** 2025-02-19

**Authors:** Boqiang Zhao, Yuhan Zhang

**Affiliations:** 1 Department of Psychology, Renmin University of China, Haidian District, No.59 Zhongguancun Avenue, Beijing, 100872 China; 2 School of Psychology, Zhejiang Normal University, Jinhua, China

**Keywords:** Chinese culture, Junzi personality, Self-reflection, Self-control, Loneliness, Subjective well-being

## Abstract

Reducing loneliness and enhancing subjective well-being are key topics in psychological research. However, few studies have explored the impact of cultural factors on loneliness and subjective well-being from a cultural perspective. Moreover, there has been few research examining the underlying mechanisms through which sociocultural factors influence individual loneliness and well-being. This study aims to explore the influence of a personality trait rooted in Chinese Confucian culture—Junzi personality—on subjective well-being among Chinese, specifically examining the mediating roles of self-reflection and self-control. A sample of 693 Chinese college students were surveyed on their Junzi personality, self-reflection, self-control, loneliness, and subjective well-being in three stages over 6 months. Correlation analysis and a multiple mediation model were conducted using SPSS 23.0 and AMOS 23.0. Results revealed that Junzi personality positively predicted subjective well-being and negatively predicted loneliness. Additionally, self-reflection, self-control, and loneliness sequentially mediated the effect of Junzi personality on subjective well-being. This study highlights the significant role of cognitive factors in the process through which sociocultural factors influence well-being.

## Introduction

### Subjective well-being and loneliness among Chinese

The pursuit of happiness has long been a central theme for humanity. Happiness or subjective well-being is generally defined as a predominance of positive over negative affect, and as satisfaction with life as a whole [9, 24]. Results from large-scale database surveys indicate that despite rapid economic and social development in China, the level of happiness among Chinese has shown an upward trend since 1990 [5]. However, the World Happiness Report 2024 indicates that China ranks 60th in subjective well-being among 137 countries and regions, suggesting significant room for improvement. As China is the most populous country in the world, enhancing well-being among Chinese is a critical issue that psychological research and theory should address. Nonetheless, psychological theories on happiness predominantly originate from Western philosophy and culture [32], raising questions about their applicability in Chinese social culture (Pan et., 2023). Therefore, it is crucial to explore theorical model that can enhance well-being among Chinese.

Reducing loneliness is an effective way to enhance subjective well-being. Loneliness is a negative emotional experience that arises when social interaction needs are unmet [19]. Basic Psychological Need Theory [41] suggests that humans inherently possess social needs—a tendency to connect with others and perceive oneself as an integral part of the social environment rather than as an isolated individual. The fulfillment of these social needs is essential for promoting psychological growth and enhancing well-being [43]. Empirical studies have shown that behaviors that satisfy social needs, such as praise, understanding, and support among friends, increase subjective well-being [8].

In China, the impact of loneliness on subjective well-being may be even more pronounced. The criteria for evaluating happiness differ across cultural contexts [34]. Hofstede's [22] Cultural Dimensions Theory posits that the impact of culture on behavior and mental status can be understood through two pivotal constructs: individualism and collectivism. Chinese who influenced by collectivism assess happiness by considering not only internal factors but also the harmony between the individual and the external environment (Oyserman, 2017). Previous studies have indicated a significant relationship between interpersonal relationships and subjective well-being among Chinese participants [23]. Therefore, this study hypothesizes that loneliness may reduce subjective well-being among Chinese.

### Junzi personality, loneliness and subjective well-being

Junzi personality, rooted in Chinese culture, may significantly influence loneliness and subjective well-being. Before exploring the impact of Junzi personality on loneliness and happiness, it is essential to briefly introduce the concept of Junzi personality. In Chinese culture, an exemplary and morally upright individual is referred to as a Junzi, characterized by traits such as benevolence, courage, and wisdom [33]. The personality traits of Junzi are seen as consummate traits pursued by every Chinese, whether in ancient or modern times (Qian, 2011). Ge et al. [16], based on the descriptions of the Junzi personality in classical Chinese texts, developed the Inventory of Junzi Personality in Confucianism after testing for its reliability and validity. The questionnaire encompasses five dimensions, including the cultivation of wisdom and courage, as well as harmonious social relations. Research has shown that Junzi personality exists across various gender and age groups [17]. These results suggest that Junzi personality is broadly applicable to various social groups in contemporary Chinese society, rather than being confined to a specific age or gender group (Qian, 2011). Furthermore, empirical research has demonstrated the differences and connections between the Junzi personality and many personality traits proposed by Western personality theories [14]. Therefore, Junzi personality can be understood as a structure shaped by the internalization of Confucian and Chinese cultural values, reflecting key characteristics of Chinese culture to some extent [16].

Both theoretical and empirical research have suggested that Junzi personality may influence the mental health of modern Chinese individuals. From the theoretical perspective, the Theory of Person-Environment Fit [45] posits that when individual-level cultural values align with cultural values at the social level, positive adaptive outcomes such as happiness increase, while negative outcomes such as depression and anxiety decrease [12, 49]. Despite the rapid development and transformation of Chinese society and culture [27], traditional cultural values, such as Confucianism, remain at the core of contemporary Chinese culture, continuing to influence the psychology and behavior of Chinese people [5],Qian, 2011). Therefore, Junzi personality, as a psychological factor rooted in traditional Chinese culture and aligned with modern Chinese cultural norms, may help reduce negative emotions and enhance psychological adaptation. From the empirical research perspective, previous findings suggested that Junzi personality is associated with many beneficial traits, which may help individuals build and maintain interpersonal relationships and enhance well-being [26]. For example, Junzi personality positively predicts mental health indicators such as interpersonal harmony and positive emotions [14, 17]. Additionally, some researchers have found that certain psychological factors rooted in traditional Chinese culture may have a negative impact on the mental health of modern Chinese individuals (Jin, 2024). Therefore, this study aims to further explore the impact of Junzi personality on the psychological well-being of Chinese individuals. Drawing from the Theory of Person-Environment Fit, we hypothesize that Junzi personality may reduce loneliness and enhance subjective well-being among Chinese.

### Junzi personality, loneliness and subjective well-being: the role of self-reflection

Although existing studies have highlighted the impact of Junzi personality on psychological well-being, few have explored the underlying mediating mechanisms. Building on previous research, self-reflection may act as a mediating mechanism through which Junzi personality influences loneliness and well-being. First, self-reflection is an effective strategy for reducing individual loneliness and enhancing overall well-being. Self-reflection is a metacognitive skill that encompasses the observation, interpretation, and evaluation of one’s thoughts, emotions, actions, and their outcomes [40]. Theories in the field of psychological counseling and therapy widely focus on the role of self-reflection in shaping an individual's sense of loneliness and overall well-being. Self-reflection-based interventions, such as Cognitive-Behavioral Therapy (CBT) and Mindfulness-Based Cognitive Therapy (MBCT), emphasize the development of self-reflection as an effective means to reduce negative emotions and improve well-being [3]. Meta-analyses have shown that self-reflection can positively predict qualities such as empathy, alleviate psychological issues like depression and anxiety, and help individuals gain more social support and subjective well-being [4],Gu et al., 2015).

Further, Chinese culture places a high value on self-reflection. For example, a classic in Chinese culture, *The Analects* (i.e., the collected sayings of Confucius and the most important text in Confucianism), states that "one should reflect on oneself several times a day, asking whether one has been diligent in working for others and honest in interactions with friends." Under the influence of such traditional culture, self-reflection is particularly important in Chinese daily lives [38]. Additionally, Chinese culture emphasizes group interests and considers intragroup harmony an essential indicator of well-being [28]. Self-reflection can reduce negative expressions in interpersonal interactions, help individuals achieve positive relationships, and maintain group harmony [36, 52]. Therefore, collectivist cultures like China encourage individuals to engage in self-reflection, leading to better interpersonal relationships and enhanced well-being [46]. Previous studies have revealed that Chinese collectivist culture encourages individuals to reevaluate and reflect on themselves, thereby reducing depression and anxiety and achieving positive social adaptation [51]. Given the central role of Junzi personality in Chinese culture, we hypothesize that Junzi personality may reduce loneliness and enhance well-being by fostering self-reflection.

### Junzi personality, loneliness and subjective well-being: the role of self-control

Self-control may also play a crucial role in how Junzi personality influences loneliness and subjective well-being. First, self-control decreases loneliness and enhances subjective well-being. The Cybernetic Control Theory posits that each person has a long-term goal representing their ideal state [6]. To achieve this goal, individuals continuously monitor the discrepancy between their current state and the desired state and reduce this discrepancy through an implementation system [25]. Self-control is a core component of this implementation system [6]. Moreover, reducing loneliness and achieving well-being are important long-term goals pursued by individuals [31]. Therefore, self-control can assist the Chinese in achieving long-term goals, such as reducing loneliness and enhancing subjective well-being. Research has found that individuals with high self-control have higher levels of interpersonal relationships, life satisfaction, and happiness in daily life [2, 30].

Moreover, the formation and development of self-control are influenced by sociocultural factors. Self-determination Theory [41] suggests that autonomy motivations such as self-integration and self-improvement enhance the development of positive abilities, such as self-control. The development of autonomy motivations is influenced by indigenous culture [13]. This theoretical perspective is also validated within Chinese Confucian culture. Confucian culture emphasizes individuals' inhibition of negative emotions during social interactions and underscores their responses to tempting stimuli [33]. Moreover, many ancient Confucian scholars believed that self-control was one of the behavioral manifestations of Junzi personality. For instance, Confucius suggested that the Junzi "even when alone, restrains selfish desires and acts with caution" [50]. Therefore, Junzi personality in Chinese culture strengthens the autonomy motivation to control oneself and enhances self-control abilities. Research indicates that Chinese participants are better able to resist responses to temptation in daily life and have higher accuracy rates in behavioral inhibition tasks than Western participants (Li et al., 2018). Based on existing research highlighting the close connection between self-control, Chinese culture, and psychological health factors, we hypothesize that self-control mediates the effect of Junzi personality on loneliness and well-being.

### Junzi personality, loneliness and subjective well-being: the role of self-reflection and self-control

Self-reflection and self-control may sequentially mediate the relationship between Junzi Personality, loneliness, and subjective well-being. First, self-reflection can influence self-control outcomes. According to the Dual-Systems Model of Self-Control [21], self-control results from the interaction of two systems: the impulsive system and the reflective system. When the reflective system suppresses the impulsive system, individuals can successfully exert self-control. Researchers have found that thoughtful deliberation helps individuals inhibit behaviors like smoking, whereas a lack of reflection often leads to impulsive actions [10, 20, 25]. The core of the reflective system involves the observation and evaluation of personal goals and current states, which is self-reflection.

Moreover, the mediating roles of self-reflection and self-control in the relationship between Junzi Personality, loneliness, and subjective well-being can be explained within the framework of Social Cognitive Theory. Social Cognitive Theory posits that human behavior is determined by the reciprocal interaction of social culture, individual cognition, and behavior [1]. According to this theory, social culture can influence psychological outcomes by affecting individual cognition [51]. Therefore, Junzi personality, as a cultural construct, may impact psychological well-being by influencing self-reflection and self-control at the cognitive level, ultimately affecting loneliness and subjective well-being.

### The present study

This study aims to explore personality and its underlying mechanisms that contribute to individual well-being from a cultural perspective. While previous studies have demonstrated the close relationship between Junzi personality, which is rooted in Chinese culture, and the psychological health of Chinese individuals [17], no research has yet examined the mediating mechanisms through which Junzi personality influences loneliness and subjective well-being. Considering the importance of self-reflection and self-control as cultural factors influencing loneliness and well-being, we hypothesize that self-reflection and self-control mediate the relationship between Junzi personality and both loneliness and subjective well-being. To test this multiple mediation model, the study employed a longitudinal design, collecting data on Junzi personality at baseline, followed by self-reflection and self-control three months later, and loneliness and subjective well-being six months after baseline.

**Fig. 1:**
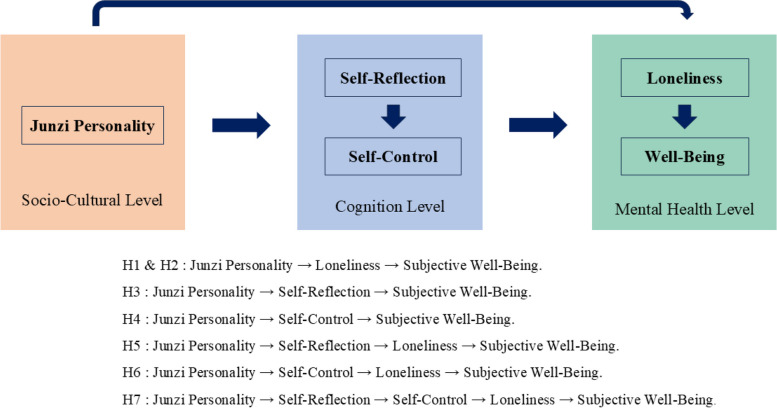
Conceptual model

Based on those mentioned theoretical and empirical studies, this study posits the following preliminary hypotheses: Junzi personality will positively predict subjective well-being among Chinese (Hypothesis 1), and the relationship between Junzi personality and subjective well-being will be mediated by loneliness (Hypothesis 2). Moreover, the relationship between Junzi personality and subjective well-being will be mediated by self-reflection and self-control (Hypothesis 3 and 4). Additionally, the relationship between Junzi personality and subjective well-being will be sequentially mediated by self-reflection and loneliness (Hypothesis 5). The relationship between Junzi personality and subjective well-being will be sequentially mediated by self-control and loneliness (Hypothesis 6). Finally, the relationship between Junzi personality and subjective well-being will be sequentially mediated by self-reflection, self-control and loneliness (Hypothesis 7). The hypothesized model is shown in Fig. 1.

## Materials and methods

### Procedures and participants

**Fig. 2:**
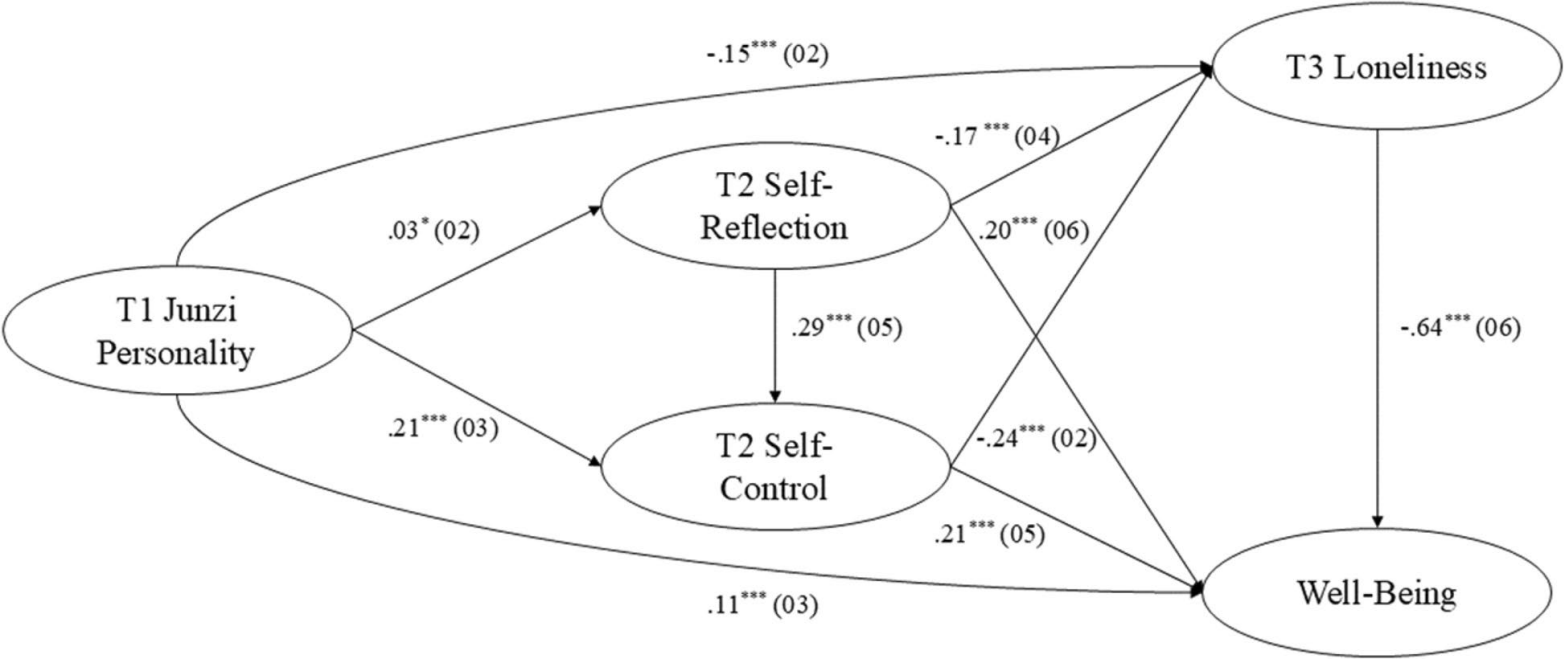
Path Coefficients for the Mediation Effect Test. ^*^p < 0.05; ^**^p < 0.01; ^***^p < 0.001

Participants from three universities in Zhejiang Province in China were randomly invited to participate in the study, following which they were directed to a web-based consent statement and (for those who agreed to participate) an online survey. Before the measurements, the participants were required to read and sign an informed consent form. All participants provided informed consent and were assured of their right to withdraw from the study at any time. Based on the principles of Monte Carlo power analysis for testing mediation effects [37] and the correlation levels among variables reported in previous studies [16], validating the hypothesized multiple-chain mediation model in this study required a sample size of 436 participants (*r* = 0.30, *α* = 0.05, 1-*β* at 0.95) Fig. 2.

Due to the limitations of cross-sectional designs in establishing causal relationships within multiple mediation models [47], this study adopted a time-lagged design to collect data on the variables in the hypothesized model across different phases. Specifically, the study took place in three stages in time over a 6-month period. In the first stage, at baseline, 746 participants completed an online questionnaire, including the Junzi personality scale and demographic variables. Three months later, 720 participants completed self-reflection and self-control scales. Six months after baseline, 693 participants completed loneliness and subjective well-being scales in the third stage. Participants who completed the first stage of the assessment received a reward of 30 RMB, and those who completed each subsequent phase were rewarded with 15 RMB per stage. All procedures adhered to the ethical guidelines established by the China Association for Psychology.

Among the remaining 693 participants, 276 were male (39.8%), 417 were female (60.2%), 257 were from urban areas (37.1%), and 436 were from rural areas (62.9%). Participants’ ages ranged from 17 to 26 years old, with a mean of 19.31 (SD = 2.65) years old. This study was preregistered before data collection and analysis (https://osf.io/cujph). This research underwent an ethical review and received approval from the university's Ethics Committee.

### Measures

#### Junzi Personality

The Inventory of Junzi Personality in Confucianism, developed by Ge et al. [16], was used to measure Junzi personality. The scale consists of 30 items rated on a 7-point Likert scale (1 = strongly disagree; 7 = strongly agree), with higher scores indicating a higher level of Junzi personality. Sample items included "I behave in accordance with the social norms," and "I am always humble and respectful to others." Cronbach's α for the scale was 0.91 in the present study. For further details of the inventory, please refer to the *Supplementary Materials*.

#### Self-reflection

The Self-Reflection and Insight Scale, developed by Grant et al. [18], was employed to assess self-reflection. The scale consists of 20 items rated on a 6-point scale (1 = strongly disagree, 6 = strongly agree). Higher scores indicated better self-reflection. Example items included "I can resist temptation well." The Cronbach's α was 0.81.

#### Self-Control

The Chinese version of the Self-Control Scale, adapted by Tan and Guo [44], was utilized to measure self-control. The scale consists of 19 items rated on a 5-point scale (1 = strongly disagree, 5 = strongly agree). Higher scores indicated better self-control. Example items included "I can resist temptation well." The Cronbach's α was 0.88.

#### Loneliness

The Loneliness Scale, developed by Russell et al. [40], was used to assess loneliness. The scale consists of 20 items rated on a 4-point scale (1 = never, 4 = always), with higher scores indicating greater loneliness. Sample items included, "Do you often feel lonely?" The Cronbach's α was 90.

#### Subjective well-being

Based on previous research [11], this study utilized the average between life satisfaction and the positive–negative affect difference as an indicator of subjective well-being. Higher scores on this measure reflect greater subjective well-being.

The Positive Affect and Negative Affect Scale, developed by Watson et al. [48], was used to assess emotional experience. This scale comprised 10 positive and 10 negative emotion words (1 = strongly disagree, 5 = strongly agree). The Cronbach's α was 0.85.

The Satisfaction with Life Scale, developed by Diener [9], was used to assess life satisfaction. The scale comprises five items rated on a 7-point scale (1 = strongly disagree to 7 = strongly agree), with higher scores indicating greater life satisfaction. Sample items include "I am satisfied with my life." The Cronbach's α was 0.85.

### Data analysis

Data analysis was carried out using SPSS 23.0 and AMOS 23.0. The analytical approach was divided into two primary phases. First, we calculated descriptive statistics and explored correlations among the variables. Second, we conducted regression analyses with Junzi personality as independent variables, and loneliness and subjective well-being as dependent variables. In this analysis, we controlled for demographic variables (age, gender, and economic status) and the respective baseline levels of adaptation. Third, we assessed the proposed multiple mediation model through structural equation modeling (SEM). The dataset for analysis consisted of the mean scores of participants across various scales, and these scores were treated as continuous data throughout the analysis. For the mediation analysis, we extracted 5000 bootstrap samples and calculated 95% confidence intervals (CI) for bootstrap (95% CI) and two-tailed probability values of < 0.05, which were considered statistically significant [51].

## Results

### Common method bias

Harman's single-factor test indicated that 21 factors had eigenvalues greater than one. The largest eigenvalue was 18.77, accounting for 16.47% of the variance. This suggests that no single factor accounted for most of the variance, indicating that the common method bias was not a significant concern [39].

### Descriptive statistics

The descriptive statistics and partial correlations among the main variables are shown in Table 1. Junzi personality exhibited positive correlations with self-reflection, self-control and subjective well-being, while displaying negative correlations with loneliness. Self-reflection exhibited positive correlations with self-control and subjective well-being, while displaying negative correlations with loneliness. Self-control exhibited positive correlations with subjective well-being, while displaying negative correlations with loneliness. Loneliness exhibited negative correlations with subjective well-being.

**Table 1:** Descriptive statistics and correlations between the variables

	*M*(*SD*)	1	2	3	4	
1. Junzi Personality	5.02(.76)	-				
2. Self-Reflection	3.59(.38)	.06	-			
3. Self-Control	2.84(.55)	.31^***^	.20^***^	-		
4. Loneliness	2.24(.46)	-.34^**^	-.21^***^	-.40^***^		
5. Subjective Well-Being	2.27(.72)	.31^**^	.24^***^	.37^***^	-.54^***^	

^*^p < .05

^**^p < .01

^***^p < .001

### Junzi personality, loneliness and subjective well-being

Results revealed that Junzi personality positively predicted subjective well-being and negatively predicted loneliness, after controlling age, gender, and socioeconomic status (subjective well-being: *B* = 0.29, *p* < 0.001; loneliness: *B* = − 0.20,* p* < 0.001).

### Test of the mediating role of self-reflection, self-control, and loneliness

We conducted a mediation analysis with AMOS. After controlling for gender, age and socioeconomic status, we selected 5000 bootstrap samples by repeating the random sampling method to establish the multiple mediation effect model (*χ*^*2*^/df = 7.98, GFI = 0.98, CFI = 0.97, RMSEA = 0.03), and the model fit well [29]. As displayed in Table 2, the total effect of Junzi personality on subjective well-being was positive (total effect = 0.29, 95%CI = [0.26, 0.36]), and the total indirect effect was significant (indirect effect = 0.18, 95%CI = [0.13, 0.24]). It explained 62.07% of the total effect. Specifically, self-reflection, self-control, and loneliness respectively mediated the relationship between Junzi personality and subjective well-being. In addition, self-reflection and self-control sequentially mediated this relationship. Self-reflection and loneliness sequentially mediated this relationship. Self-control and loneliness also sequentially mediated this relationship. Self-reflection, self-control and loneliness also sequentially mediated this relationship. Significant mediation was observed, with detailed path coefficients and the sizes of mediation effects presented in Table 3.

**Table 2:** Estimates and 95% CIs for Indirect Effects

Pathways	Estimate(*B*)	95%CI	
**Total indirect effect**	.18^***^	.13	.24
**Specific indirect effect**			
Junzi Personality → Self-Reflection → Subjective Well-Being	.01^*^	.00	.02
Junzi Personality → Self-Control → Subjective Well-Being	.04^***^	.02	.17
Junzi Personality → Loneliness → Subjective Well-Being	.09^***^	.06	.13
Junzi Personality → Self-Reflection → Self-Control → Subjective Well-Being	.01^*^	.00	.01
Junzi Personality → Self-Reflection → Loneliness → Subjective Well-Being	.01^*^	.00	.01
Junzi Personality → Self-Control → Loneliness → Subjective Well-Being	.03^*^	.02	.05
Junzi Personality → Self-Reflection → Self-Control → Loneliness → Subjective Well-Being	.01^*^	.00	.01

^*^p < .05

^**^p < .01

^***^p < .001

**Table 3:** Testing the mediating model

	M1: Self-Reflection		M2: Self-Control		M3: Loneliness		Y: Well-Being	
	*B(SE)*	95% CI	*B(SE)*	95% CI	*B(SE)*	95% CI	*B(SE)*	95% CI
X: Junzi personality	.03^*^(.02)	[.00, .07]	.21^***^(.03)	[.16, .25]	-.15^***^(.02)	[-.26, -.09]	.11^***^(.03)	[.05, .17]
M1: Self-Reflection	-	-	.29^***^(.05)	[.19, .39]	-.17^***^(.04)	[-.26, .09]	.20^***^(.06)	[.07, .32]
M2: Self-Control	-	-	-	-	-.24^***^(.02)	[-.30, -.18]	.21^***^(.05)	[.12, .30]
M3: Loneliness	-	-	-	-			-.64^***^(.06)	[-.75, -.53]
U1: Gender	-.11^***^(.03)	[-.17, -.05]	.10^*^(.04)	[.02, .18]	-.01(.03)	[-.06, .07]	-.04(.05)	[-.14, .05]
U2: Age	.02^**^(.01)	[.01, .03]	-.01(.01)	[-.03, .00]	.01(.01)	[-.01, .02]	.01(.01)	[-.01, .02]
U3: Economic status	-.001(.01)	[-.02, .02]	.01(.01)	[-.01, .04]	-.03^***^(.01)	[-.05, -.01]	.01(.01)	[-.02, .04]
	*R*^*2*^ = .04	*F* = 7.54^***^	*R*^*2*^ = .14	*F* = 22.38^***^	*R*^*2*^ = .24	*F* = 37.04^***^	*R*^*2*^ = .11	*F* = 20.23^***^

*B* Estimate, *SE* Robust standard error, *95% CI* 95% confidence intervals

^*^p < .05, **p < .01, ***p < .001

## Discussion

Overall, this study found four main results: (1) Junzi personality negatively predicted loneliness and positively predicted subjective well-being; (2) Self-reflection mediated the relationship between Junzi personality and loneliness as well as subjective well-being; (3) Self-control also mediated the effect of Junzi personality on loneliness and subjective well-being; (4) Self-reflection and self-control collectively mediated the relationship between Junzi personality and both loneliness and subjective well-being. Next, we will discuss these four key findings. Based on these results, we further explore the theoretical and practical implications of this study.

The results support hypotheses 1 and 2, indicating that Junzi personality positively predicts subjective well-being and that loneliness mediates this process. Previous studies have also found similar results, demonstrating the negative impact of Junzi personality on negative emotions such as depression and anxiety, and its positive impact on social support, psychological adaptation, and well-being [15, 16]. These results can be explained from two perspectives. First, the Cultural Norm Model and Theory of Person-Environment Fit suggest that culture is the primary factor in forming the standards for evaluating well-being [35, 45]. The degree to which individuals act according to social norms or cultural standards determines their level of well-being (Lu, 2003). The concept of Junzi is central to Confucian culture, reflecting Confucian scholars' ideal vision of an individual's moral character [33]. Therefore, as a core component of Confucian and Chinese culture, Junzi personality aligns individual behavior with cultural norms, enhancing well-being. Second, according to the Basic Psychological Needs Theory, every person has basic psychological needs such as social needs, and the satisfaction of these needs is a prerequisite for achieving a healthy psychological state and well-being [41, 43]. The generation of basic psychological needs is also influenced by culture. Since Chinese culture places great importance on interpersonal relationships and in-group harmony, satisfying social relationships is particularly crucial for Chinese [34]. Therefore, individuals with a Junzi personality in China are able to build good social relationships, reduce loneliness, and experience greater well-being.

The results of this study support Hypothesis 3 and Hypothesis 5. The findings indicate that self-reflection mediates the effect of Junzi personality on loneliness and subjective well-being. The reasons for the mediating role of self-reflection between Junzi personality and well-being may be as follows. First, Confucian culture emphasizes the cultivation of self-reflection abilities. Confucianism suggests a hierarchical sequence of self-fulfillment goals, including self-cultivation, family harmony, and world peace [7]. Self-reflection aids individuals in enhancing self-cultivation, which is the primary goal for achieving other long-term objectives (Qian, 2011). Therefore, Confucian culture advocates for individuals to engage in self-reflection. Moreover, self-reflection can enhance well-being. According to Cognitive-Behavior Therapy (CBT), accurate observation and evaluation of one's state can effectively alleviate immediate negative experiences and improve long-term psychological adaptation and well-being [4]. Empirical studies have shown that prolonged self-reflection training increases psychological resilience and resources, reduces depression and anxiety, and enhances well-being [36].

The results of this study support Hypotheses 4 and 6, indicating that self-control mediates the influence of Junzi personality on loneliness and subjective well-being. Previous research has similarly demonstrated that self-control plays a mediating role in the relationship between Chinese cultural values and psychological health [16]. The mediating role of self-control can be elucidated through both contemporary psychological theories and Confucian philosophical thought. On the one hand, according to Cybernetic Control Theory [6], although self-control may deplete psychological resources, it effectively resists immediate temptations and helps achieve long-term adaptive goals. Furthermore, Self-determination Theory posits that macro-level sociocultural factors enhance individual autonomous motivation, thereby fostering the development of beneficial qualities such as self-control [42]. Thus, internalizing Junzi personality shaped by Chinese culture enhances self-control, thereby contributing to long-term goals such as reducing loneliness and increasing well-being (Qian, 2011). On the other hand, Confucianism underscores the significance of self-control in achieving individual goals. Confucian thought highlights that the characteristics of Junzi can restrain personal desires [33]. Therefore, by integrating Eastern and Western cultural perspectives, Junzi personality can enhance self-control, thereby reducing loneliness and improving well-being.

Ultimately, the results of this study support Hypothesis 7, indicating that self-reflection, self-control, and loneliness collectively mediate the relationship between Junzi personality and subjective well-being. This multiple mediation effect can be simplified to a process where sociocultural factors influence psychological outcomes through individual cognition, as explained by Social Cognitive Theory. According to Social Cognitive Theory, individual cognition serves as the bridge through which sociocultural factors affect psychological outcomes [1]. As previously discussed, we propose that Junzi personality represents a cultural-specific trait formed by the internalization of Confucian and Chinese cultural values, reflecting the broader societal Chinese culture [15]. Self-reflection and self-control are central functions of cognition, with self-reflection underpinning self-control functions [21, 25]. Low levels of loneliness can lead to higher levels of well-being, with both being indicators of a healthy psychological state [8]. Therefore, the societal Junzi personality can impact cognitive processes such as self-reflection and self-control, thereby influencing psychological outcomes such as loneliness and well-being.

Given that existing theories on promoting well-being are predominantly derived from Western cultural perspectives, this study aims to explore the influence of cultural factors on well-being from the standpoint of Eastern Confucian culture and to examine the mediating mechanisms involved. The study innovatively identifies that a culturally specific personality trait—Junzi personality—can reduce loneliness and enhance well-being among Chinese. This finding supports the Theory of Person-Environment Fit [45] and the Cultural Norm Model [35], indicating that the alignment between individual beliefs and societal values contributes to achieving a healthy psychological state. Furthermore, this study reveals that self-reflection and self-control are multiple mediators in the relationship between Junzi personality and loneliness and well-being. These results support and extend Social Cognitive Theory [1] and Self-Determination Theory [41], demonstrating that the impact of sociocultural factors on individual psychology and behavior is mediated by individual cognition, with cognitive factors themselves exerting mutual influences.

By elucidating the mechanisms through which cultural factors influence loneliness and subjective well-being, this study provides valuable insights for developing psychological well-being interventions. For instance, based on the findings of this study and the Theory of Person-Environment Fit, governments should promote their nation's excellent traditional culture, enhance individuals' identification with and confidence in their own culture, and help individuals develop values, beliefs, and personality traits consistent with their national culture. These measures are likely to contribute to increased well-being. Furthermore, individuals should focus on developing abilities such as self-reflection and self-control to achieve long-term goals, ultimately enhancing their well-being. Such positive qualities can be fostered through a deeper engagement with esteemed traditional culture.

## Conclusion

In summary, this study found that the relationship between Junzi personality and subjective well-being could not only be mediated by self-reflection, self-control and loneliness in an independently mediated manner but by self-reflection, self-control and loneliness in a sequentially mediated manner. Our study suggests that promoting self-reflection, self-control and reducing loneliness may contribute to a promotion of subjective well-being among Chinese. This study also has certain limitations. First, while Junzi personality is considered a personality within Chinese culture, it originates from Confucianism. Future research could examine whether Junzi personality also exists as a general personality trait in individuals from other East Asian countries and even Western countries and explore its psychological and behavioral effects on individuals from other countries. Second, the study sample is primarily limited to university students, which may restrict the generalizability of the findings. Future research could extend the sample to other groups to explore the generalizability of the results. Lastly, this study collected data using self-reported questionnaires, which may be subject to self-report biases. Future research could incorporate objective measures for validation.

## Data Availability

Supplementary materials and data are publicly available at https://osf.io/wxgvj/
